# Validation of Indocyanine Green-Methylene Blue Dye in the Lymphedema Rat Tail Model

**DOI:** 10.3390/biomedicines14020324

**Published:** 2026-01-30

**Authors:** Joon Seok Lee, Woosung Jang, Hyun Geun Cho, Jun Sik Kim, Sang Hyun An, Sungdae Na, Byeongju Kang, Jeeyeon Lee, Ho Yong Park, Jeong Yeop Ryu, Kang Young Choi, Jung Dug Yang, Ho Yun Chung, Jeongsoo Yoo, Wonchoul Park

**Affiliations:** 1 Department of Plastic and Reconstructive Surgery, School of Medicine, Kyungpook National University, Daegu 41566, Republic of Korea; 2 Preclinical Research Center, Daegu-Gyeongbuk Medical Innovation Foundation (K-MEDI Hub), Daegu 41061, Republic of Korea; 3 Department of Biomedical Engineering, Kyungpook National University Hospital, Daegu 41404, Republic of Korea; bluepoison14@knu.ac.kr; 4 Department of Surgery, School of Medicine, Kyungpook National University, Daegu 41566, Republic of Korea; 5 BK21 Four Knu Convergence Educational Program, Department of Molecular Medicine, School of Medicine, Kyungpook National University, Daegu 41944, Republic of Korea; 6 Capabioscience Co., Ltd., Guri-si 11901, Republic of Korea

**Keywords:** lymphedema, indocyanine green-methylene blue, lymphatic flow

## Abstract

**Background/Objectives:** Lymphedema is characterized by edema; in severe cases, skin changes and ulceration significantly impair patients’ quality of life. Although several experimental rodent models for lymphedema have been established, a reproducible and practical model remains essential for evaluating new therapeutic and imaging agents. This study aimed to establish a lymphedema animal model and to evaluate the efficacy of a newly synthesized dual-mode imaging reagent as a potential alternative to indocyanine green (ICG). **Methods:** Eleven Sprague-Dawley rats were classified into two groups. Full-thickness skin excision was performed on the tails of nine rats to induce lymphedema; two rats served as controls. Five rats received ICG injections for 1 week postoperatively, while the remaining six rats were administered tail injections of chemically synthesized indocyanine green-methylene blue (ICG-MB) reagent. Lymphatic flow was photographed using a SPY camera. After euthanasia, tail segments were analyzed by microcomputed tomography (micro-CT) to measure volume and by hematoxylin–eosin staining for histological evaluation. **Results:** On postoperative day 7, lymphatic flow was confirmed in the ICG-MB group using the SPY Elite^®^ fluorescence imaging system. On micro-CT scans, the preoperative rat tail volume was 3992.72 ± 144.80 mm^3^. Rat tail volume was 5216.71 ± 1131.88 and 4614.76 ± 468.29 mm^3^, respectively, at 1 and 2 weeks after lymphedema was induced. Histology revealed lymphocyte infiltration, inflammatory reaction, and thickened subcutaneous adipose tissue, with no significant difference between groups. **Conclusions:** The rat tail lymphedema model proved valuable for studying lymphedema pathology and diagnostic agents. The ICG-MB reagents demonstrate stable performance and favorable biocompatibility.

## 1. Introduction

Lymphedema is a disease characterized by a decline in lymphatic transport capacity due to damage or dysfunction of the lymphatic system, causing abnormal accumulation of the lymph in the interstitial tissue of an affected body part [1,2,3]. Despite extensive research on the lymphatic system, a universally reliable treatment method for lymphedema remains under debate. The pathophysiology of lymphedema initially involves edema caused by fluid accumulation, and with disease progression, there is increased adipose tissue deposition and fibrotic tissue formation, leading to tissue enlargement. This enlargement further impairs lymphatic function and promotes the accumulation of protein-rich fluid, increasing susceptibility to infection. In severe cases, it may cause skin changes and ulcers [4,5].

Lymphedema can be categorized into primary and secondary types [6]. Primary lymphedema refers to congenital cases without a known cause, whereas secondary lymphedema develops when a previously normal lymphatic system is damaged by infection, surgery, trauma, tumors, radiation, or other factors. The most common type of secondary lymphedema develops after treatment for malignant tumors. Upper extremity lymphedema commonly develops following surgical treatment for breast cancer, whereas lower extremity lymphedema frequently occurs after surgery for gynecological cancers. With the rising rate of early diagnosis for various malignant tumors, the incidence of secondary lymphedema has steadily increased recently and research into treatments is on-going [7,8].

Recently, advanced imaging technologies have been developed to visualize fluorescent dyes preoperatively or intraoperatively using specialized equipment, enabling precise real-time observation of lymphatic fluid flow and vessels [9,10]. Among these technologies, the most widely used reagent is indocyanine green (ICG) fluorescent dye, which emits at wavelengths between 775 and 830 nm and can be detected using a near-infrared camera. This technique is valuable for assessing the flow and extent of lymphatic fluid. Clinically, ICG has demonstrated utility across various medical and surgical fields, including angiography, cerebral blood flow evaluation, liver function assessment, and sentinel lymph node biopsy in breast cancer. However, this approach is limited by the requirement for specialized and costly fluorescent imaging equipment, since the dye is invisible to the naked eye [11,12]. Although surgical advances have been made, helping divert lymphatic fluid into surrounding veins or enabling vascularized lymph node transfer, these methods require highly advanced and extremely costly equipment capable of visualizing ICG through super-microsurgery.

Therefore, we hypothesized that combining ICG, which emits near-infrared wavelengths, with methylene blue (MB, a reagent still widely used for visual inspection) could yield a dual-mode agent capable of fluorescent and visual detection, enabling preoperative diagnosis and surgical site visualization. This reagent could also be visually observed and potentially serve as a probe for lymphedema surgery or other procedures. We proposed that a reagent combining fluorescent and visually detectable properties, enabling observation without the need for costly near-infrared equipment, could expand its use in preoperative and intraoperative treatments.

## 2. Materials and Methods

### 2.1. Experimental Materials

#### Sprague-Dawley (SD) Rats

Eleven female SD rats aged 10 weeks and weighing over 200 g were bred in the Preclinical Research Center of the Daegu Gyeongbuk Advanced Medical Industry Promotion Foundation (K-Medi Hub). Twenty-two female SD rats, weighing 200–300 g, were used in the experiment. Upon arrival, the animals were subjected to quarantine and a 1-week acclimatization period. All animals were carefully examined to ensure they exhibited no abnormal symptoms before the experiment. Each animal was individually marked on the tail with a red permanent marker upon arrival, and corresponding identification cards were attached to their housing cages. During the monitoring period, animals were marked with a blue permanent marker on the tail to indicate group assignment. Labels were attached to the housing cages, specifying the Institutional Animal Care and Use Committee (IACUC), the principal investigator, species, strain, sex, date of birth, experimental period, date of receipt, individual number, and notes.

The animals were separated into two groups on the last day of acclimatization to create a tail lymphedema model: two rats were designated as controls, while nine were assigned for lymphedema induction. All rats were fed ad libitum with R+40RMM-10 rodent feed. The environment in the animal rearing room was kept at a temperature, humidity, ventilation rate, and illumination of 22 ± 1 °C, 50 ± 10%, 10–25 times/h, and 150–300 Lux, respectibely, under a 12 h day/night cycle. The rearing cages were cleaned and sterilized using an automatic washer and autoclave, respectively. Rodent feed was sourced from WOOJUNGBIO, Inc. (Hwaseong-si, Republic of Korea) and provided ad libitum after verifying its quality. Purified reverse osmosis water, sourced from the Daegu Metropolitan City supply, was provided ad libitum. Water quality was tested by the Daegu Research Institute of Public Health and Environment across 58 parameters to screen for potential contaminants. All animal experiments were conducted following approval from the IACUC of K-Medi Hub (Approval date: 6 October 2022; IACUC Approval No. KMEDI-22101101-00).

### 2.2. ICG-M Synthetic Reagent

**Figure 1:**
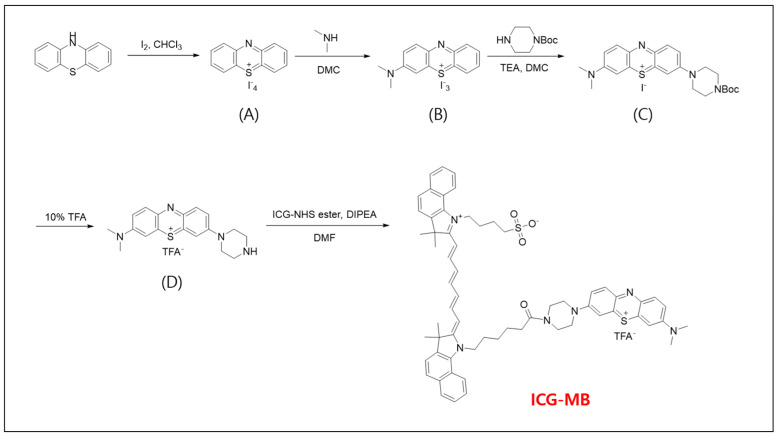
Synthesis of indocyanine green-methylene blue (ICG−MB). (**A**) Phenothiazin−5−ium tetraiodide hydrae, (**B**) 3−Dimethylaminophenothiazin−5−ium triiodide, (**C**) 3−[4−(tert-butoxycarbonyl)piperazin−1−yl] −7−(dimethylamino)phenothiazin−5−ium iodide (**D**) 3−(piperazin−4−ium−1−yl) −7−(dimethylamino)phenothiazin−5−ium 2∙TFA. The ICG−MB reagent was obtained through a five-step synthesis starting from phenothiazine, with isolated yields of 95% (**A**), 55% (**B**), 75% (**C**), 99% (**D**), and 83% for the final ICG–MB product.

The ICG-MB reagent was synthesized in five steps (Figure 1).

(1) Phenothiazin-5-ium Tetraiodide Hydrate (A)

A solution of phenothiazine (2.13 g, 11 mmol) in CHCl_3_ (70 mL) was stirred at 0 °C and added over 30 min with a solution of I_2_ (8.38 g, 33 mmol) in CHCl_3_ (70 mL). The mixture was stirred at 5 °C for an additional 30 min. The resulting precipitate was filtered, washed with CHCl_3,_ and dried, yielding compound 1 as a dark-purple solid (7.25 g, 95%). ^1^H NMR (Acetone-d_6_): δ 8.08 (m, 2H), 7.98 (m, 2H), and 7.71 (m, 4H).

(2) 3-Dimethylaminophenothiazin-5-ium triiodide (B)

A solution of phenothiazin-5-ium tetraiodide hydrate (6.15 g, 8.5 mmol) in dichloromethane (200 mL) was treated dropwise with a solution of dimethylamine in methanol (2 M, 16.99 mL, 8.5 mmol) over 6 h using a syringe pump. The reaction mixture was allowed to stand overnight at room temperature. The resulting precipitate was filtered, washed with CH_2_Cl_2_, and dried. The product was recrystallized from methanol to afford compound 2 as a dark-blue solid (2.91 g, 55%). ^1^H NMR (DMSO-d_6_): δ 8.23 (dd, 1H), 8.17 (dd, 1H), 8.11 (d, 1H), 8.04 (dd, 1H), 7.99 (d, 1H), 7.84 (m, 2H), 3.65 (s, 3H), and3.60 (s, 3H).

(3) 3-[4-(tert-butoxycarbonyl) piperazin-1-yl]-7-(dimethylamino) phenothiazin-5-ium iodide (C)

A solution of 3-dimethylaminophenothiazinium triiodide (1 g, 1.6 mmol) in dichloromethane (50 mL) was added dropwise a solution of triethylamine (0.33 mL, 2.4 mmol) in dichloromethane (50 mL). After stirring for 5 min, a solution of 1-Boc-piperazine (0.9 g, 4.8 mmol) in dichloromethane (20 mL) was added over 30 min. The mixture was stirred overnight at room temperature and washed with water (3 × 250 mL). The organic layer was dried over MgSO_4,_ and the solvent was evaporated at reduced pressure, not exceeding a water bath temperature of 40 °C. The crude material was purified by silica gel chromatography using dichloromethane/ethanol 10:1 as the eluent, affording purple glimmering crystals (0.67 g, 75%). ^1^H NMR (DMSO-d_6_): δ 7.85 (m, 2H), 7.62 (1H, m), 7.58 (m, 4H), 3.86 (m, 4H), 3.54 (m, 4H), 3.37 (s, 6H), and 1.43 (s, 9H).

(4) 3-(piperazin-4-ium-1-yl)-7-(dimethylamino) phenothiazin-5-ium 2∙TFA (D)

The 3-[4-(tert-butoxycarbonyl) piperazin-1-yl]-7-(dimethylamino) phenothiazin-5-ium iodide (3) (0.5 g, 0.91 mmol) was dissolved in dichloromethane (4 mL). Next, 10% TFA solution in dichloromethane (2 mL) was added dropwise, and the reaction mixture was stirred for 5 h at room temperature. The product was precipitated by adding diethyl ether (50 mL), and subsequently, the resultant precipitate was filtered, washed with diethyl ether, and allowed to air dry, yielding 2 as a dark-blue solid (0.54 g, 99%). ^1^H NMR (DMSO-d_6_): δ 9.05 (m, 2H), 8.02 (m, 2H), 7.64 (m, 2H), 3.99 (m, 4H), 3.47 (s, 6H), and 3.32 (m, 4H).

(5) ICG-MB

ICG-NHS ester (19 mg, 23 μmol) was added to a stirred mixture solution of compound 4 (14 mg, 1.7 μmol) and DIPEA (20 μL, 0.12 mmol) in DMF (5 mL) and the mixture was stirred for 2 h at room temperature. The solvent was evaporated under reduced pressure, and the residue was recrystallized from methanol/diethyl ether to yield ICG-MB as a dark-blue solid (20 mg, 83%). ^1^H NMR (DMSO-d_6_): δ 7.85 (m, 2H), 7.62 (1H, m), 7.58 (m, 4H), 3.86 (m, 4H), 3.54 (m, 4H), 3.37 (s, 6H), and 1.43 (s, 9H). MS-ESI m/z: 1037.8 (M^+^).

The overall synthetic route afforded ICG–MB in 83% isolated yield in the final coupling step, following intermediates A–D with yields of 95%, 55%, 75%, and 99%, respectively.

### 2.3. Experimental Methods

#### 2.3.1. Establishment of Experimental Models

**Figure 2:**
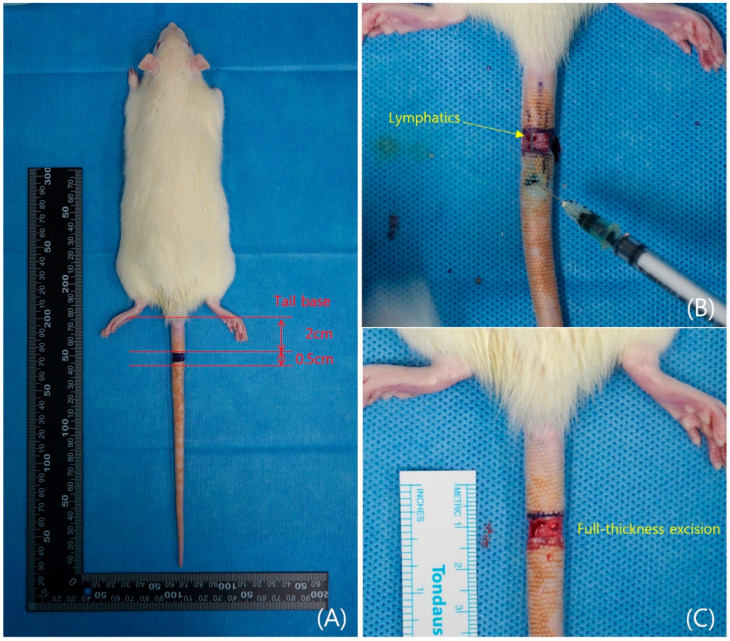
Surgical procedure for secondary lymphedema in a rat tail model. (**A**) Pre-operative design. (**B**) Lymphatic vessels after injection of indocyanine green (ICG). (**C**) Full-thickness skin removal was performed on a 20 mm area from the rat tail base.

All animals were subcutaneously administered gentamycin (20 mg/kg) and meloxicam (1.2 mg/kg) as prophylactic antibiotic and analgesic agents, respectively. General anesthesia was induced via intraperitoneal injection of 30 mg/kg Zolazepam (Zoletil^®^, Virbac, Seoul, Republic of Korea) and 10 mg/kg Xylazine (Rompun^®^, Bayer, Leverkusen, Germany). The experimental animals were monitored for respiration and anesthesia status, and subsequently, their tails were disinfected three times with alcohol and a 10% povidone solution. To disrupt the superficial lymphatic vessels, a 5 mm-wide full-thickness section of skin was excised 20 mm from the base of the tail in all rats. To remove the deep lymphatic vessel running parallel to the lateral tail vein, ICG (Diagnostic Green LLC, Farmington Hills, MI, USA) was injected subcutaneously to visualize the vessel’s course. The deep lymphatic vessel was carefully excised using a No. 15 surgical blade. Care was taken to avoid vascular injury during lymphatic excision. The incision site was covered with 3M^TM^ Tegaderm^TM^ to maintain moisture and prevent infection, with dressings changed daily (Figure 2).

#### 2.3.2. Observations of Clinical Symptoms and Measurements of Tail Volume

From the start of the experiment until its conclusion at 2 weeks post-surgery, animals were monitored daily for general appearance, water and feed intake, and any abnormal clinical signs.

### 2.4. Experimental Evaluations

#### 2.4.1. Visual Evaluation

For visual evaluation after incision, close-up photographs of the surgical site on the experimental animal were taken using a digital camera (Sony A7 III, Sony, Tokyo, Japan). Following euthanasia, the tails were incised and photographed postmortem before tissue collection. After the experiment, we performed a visual evaluation for lymphedema.

#### 2.4.2. Acquisition of Lymphatic Flow Images

Overall, 10 μL of ICG reagent was injected subcutaneously at a point 30 mm from the tail tip in five rats, and 10 μL of the prepared ICG-MB reagent was injected at the same location in the remaining six rats. Five minutes after injection, lymphatic flow at the surgical site was imaged using the SPY Elite^®^ Fluorescence Imaging System (Stryker Corp., Portage, MI, USA) at the Preclinical Research Center of K-Medi Hub.

#### 2.4.3. Measurement of Tail Volume

On postoperative day (POD) 7 and 14, the tails of 11 rats were excised 25 mm from the base and imaged using the Quantum FX micro-computed tomography (CT) system (PerkinElmer, Waltham, MA, USA) at the Preclinical Research Center of K-Medi Hub. The X-ray source was operated at 90 kVp and 180 μA. Tail volume was quantified using Analyze 12.0 software.

#### 2.4.4. Histological Evaluation

For histopathological evaluation, tissue samples were collected from the ICG and ICG-MB groups before lymphedema induction and at POD7 and 14. Approximately 5 mm-thick sections were excised from locations 3 and 6 cm distal to the surgical site. Each tissue was trimmed, dehydrated in alcohol using a tissue processor, pretreated to facilitate paraffin penetration, and subsequently fixed in paraffin in a cassette using an embedding machine to create tissue blocks. Tissue sections were subjected to hematoxylin and eosin (H&E) staining for histopathological analysis. Stained tissue sections were examined using an Olympus CX23 light microscope (Olympus Corporation, Tokyo, Japan) to compare histological differences between the ICG and ICG-MB groups, including subcutaneous adipose tissue thickness.

### 2.5. Statistical Analysis

All measurements were analyzed using IBM SPSS Statistics for Windows, version 22.0 (IBM Corp., Armonk, NY, USA). Results are expressed as mean ± standard deviation. A paired *t*-test was used to test for statistical significance in the experimental group, with a significance level set at less than 5% (*p* < 0.05).

## 3. Results

Rats were weighed daily to monitor growth and identify any deviations from normal weight gain. No deaths occurred during the experiment. Other than a few inflammatory findings due to lymphedema, the rats grew normally with no significant complications observed.

### 3.1. Visual Findings

**Figure 3:**
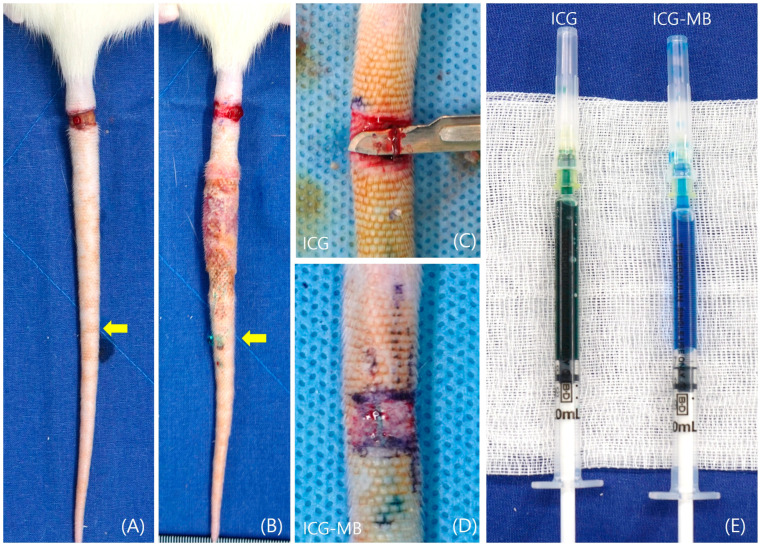
Postoperative day 7 findings in the lymphedema rat tail. (**A**) Lymphedema-induced rat tail. (**B**) Inflammation was identified in the lymphedema rat tail. (**C**,**D**) Immediate gross appearance of lymphatic flow after ICG and ICG-MB injection. (**D**,**E**) ICG-MB (Right) & ICG (Left), (yellow arrow; ICG or ICG-MB injection point). ICG, indocyanine green; ICG-MB, indocyanine green-methylene blue.

During the observation period after lymphedema induction, two rats exhibited visual signs of lymphedema-associated inflammation. No external wound infections or other complications were observed. Rats that underwent full-thickness skin excision displayed more pronounced lymphedema (Figure 3).

### 3.2. Imaging of Lymphatic Flow

**Figure 4:**
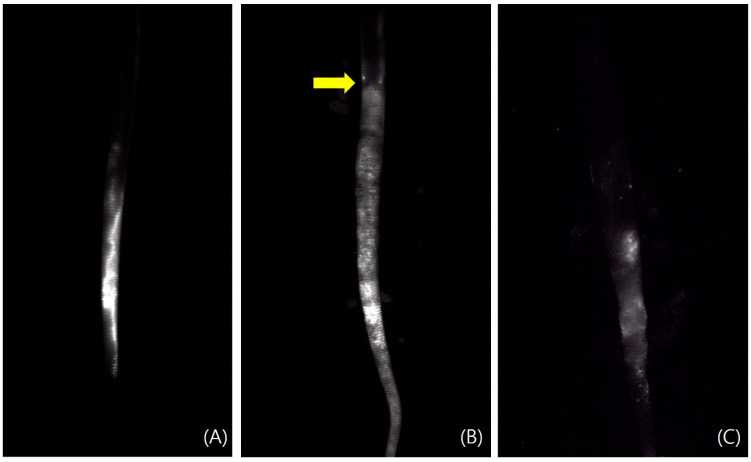
SPY fluorescence after injecting indocyanine green (ICG) and indocyanine green-methylene blue (ICG-MB). (**A**) ICG was injected into the rat tail of the control group and imaged with a SPY camera. Normal lymphatic flow is examined. (**B**) The image taken after injecting ICG into the rat’s tail that caused lymphedema. Blocked lymphatic flow is assessed. (Yellow arrow). (**C**) The image taken after injecting ICG-MB into the rat tail that caused lymphedema.

In weeks 1 and 2 post-lymphedema induction, five rats were administered ICG reagent, and the remaining six received the ICG-MB reagent. Lymphatic flow was imaged using the SPY Elite^®^ Fluorescence Imaging System (Stryker Corp., Portage, MI, USA) at the Preclinical Research Center of K-Medi Hub. In control animals, the ICG and ICG-MB enabled clear visualization of lymphatic flow. In contrast, the nine experimental rats exhibited a marked disruption of lymphatic flow. Although fluorescence intensity was reduced in the ICG-MB group compared to ICG, lymphatic fluid accumulation remained clearly visualized on the SPY camera (SPY Elite Fluorescence Imaging System, Stryker Corp., Portage, MI, USA) after administering a higher reagent dose (Figure 4).

### 3.3. Measurement of Tail Volumes

**Figure 5:**
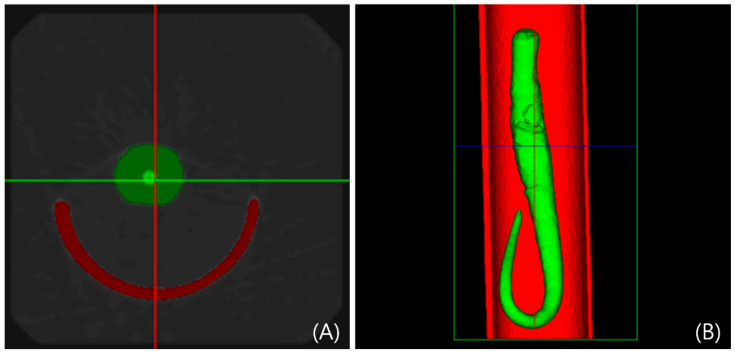
Rat tail volume measurement using micro-computed tomography (micro-CT). Tail was photographed through Quantum FX micro-CT (Perkin Elmer, Waltham, MA, USA). The tail volume was measured using Analyze 12.0. (**A**) Coronal view findings. (**B**) Axial view findings.

On POD 7 and 14, a 25 mm tail segment was excised from each of the 11 rats and imaged using the Quantum FX micro-CT system (Perkin Elmer, Waltham, MA, USA) in the Preclinical Research Center of K-Medi Hub. The preoperative tail volume of rats was 3992.72 ± 144.80 mm^3^. One week after lymphedema induction, the tail volume became 5216.71 ± 1131.88 mm^3^, showing a significant increase (* *p* < 0.05) (Figure 5).

### 3.4. Histological Findings

**Figure 6:**
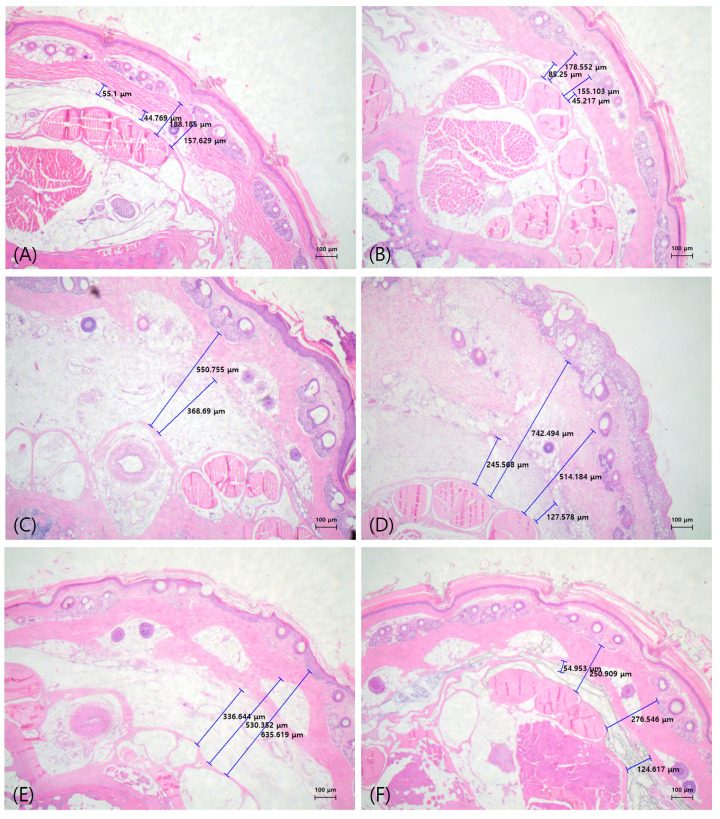
Representative photographs of hematoxylin & eosin (H&E) staining. Histological findings of the rat tails stained with H&E. (**A**) Normal rat tail model injected with indocyanine green (ICG). (**B**) Normal rat tail model injected with indocyanine green-methylene blue (ICG-MB). (**C**) Findings in the rat tails injected with ICG 1 week postoperatively. H&E staining revealed thickening of the subcutaneous tissue layer postoperatively. A significant increase was observed in adipose tissue. (**D**) Findings in rat tails injected with ICG-MB 1 week postoperatively. (**E**) Findings in rat tails injected with ICG 2 weeks postoperatively. H&E staining revealed thickening of the subcutaneous tissue layer after surgery. A significant increase was observed in adipose tissue. (**F**) Findings in rat tails injected with ICG-MB 2 weeks postoperatively. No significant difference was observed in the histological findings of the group injected with ICG and ICG-MB.

After euthanasia, tissue samples were collected at 30 and 60 mm distal to the surgical site and sectioned into 5 mm thick slices. Samples were subjected to H&E staining and were subsequently examined under a microscope. Following lymphedema induction, lymphocyte numbers increased along with an enhanced inflammatory response. The subcutaneous adipose layer of rats in the experimental group was approximately three- to four-fold thicker than that of those in the control group. Importantly, no significant histological differences were observed between rats injected with ICG and those injected with ICG-MB compared to the control group at weeks 1 and 2 (Figure 6).

## 4. Discussion

Lymphedema is a condition characterized by the accumulation of lymphatic fluid in interstitial tissues due to reduced lymphatic transport capacity resulting from damage or dysfunction of the lymphatic system. The prevalence of lymphedema has been increasing in recent years. Initially, edema occurs, followed by the accumulation of adipose and fibrous tissue, which leads to progressive tissue enlargement. In severe cases, skin changes and ulceration may develop over time [13,14,15,16,17,18,19,20,21,22,23,24].

Several diagnostic approaches are available for lymphedema. Recently, technologies have advanced to allow for clear real-time observation of lymphatic fluid flow and lymphatic vessels through instruments intraoperatively or preoperatively, using fluorescent dyes that emit specific wavelengths [25]. Among these, ICG is the most widely used fluorescent dye. ICG has been proven effective in various clinical and surgical fields, including angiography, cerebral blood flow measurement, liver function assessment, and sentinel lymph node biopsy in breast cancer. In addition to ICG, MB and indigo carmine are frequently used as staining reagents in clinical practice. MB is commonly used in procedures involving urinary tract disorders, including as urinary calculi, cystitis, and urethritis, as well as in sentinel lymph node biopsy for breast cancer. Indigo carmine has also been used in sentinel lymph node biopsy, detection of amniotic fluid leakage, and identification of the urinary tract [26,27].

One major limitation of ICG is that its emission falls within the near-infrared range, beyond the visible spectrum. Consequently, the lymphatic flow cannot be directly visualized during surgery requiring expensive equipment for tracking lymphatic fluid [28,29]. To overcome this limitation of ICG, we developed a reagent that can be detected both through fluorescence imaging and direct visual inspection due to its intrinsic color. First, we selected MB and indigo carmine as candidates because their suitable wavelengths for chemical coupling to ICG and established clinical use [30]. Among the two, indigo carmine posed challenges for synthesis due to its relatively large molecular weight. Subsequently, its increased molecular weight could cause problems during synthesis or in biological systems after synthesis. In contrast, MB has a relatively smaller molecular weight than indigo carmine; is more predictable to synthesize; and has been used stably for visualization and orientation in various medical devices for a long time. Furthermore, combining MB and ICG has demonstrated a 100% detection rate in axillary lymph node mapping for early-stage breast cancer. Therefore, we decided to synthesize a combination of MB and ICG. The reagent was synthesized via a five-step chemical process (Figure 1) [31].

We established a lymphedema model by surgically excising and ligating the lymphatic vessels in the tails of SD rats. The control group received ICG, while the experimental group was administered ICG-MB, our synthesized dual-detection reagents. Lymphatic detection was evaluated using the SPY Elite^®^ Fluorescence Imaging System (Stryker Corp., Portage, MI, USA), a platform widely adopted in clinical practice. Changes in the volume of the tails of rats with lymphedema induction were calculated using micro-CT, and histological changes were also examined.

Visual evaluation revealed inflammatory changes associated with lymphedema in two of the rats. This presentation resembles the clinical features of advanced lymphedema, which is frequently complicated by cellulitis. However, no overt signs of external wound infections or other postoperative complications were noted in the affected animals. Furthermore, animals where lymphatic vessels were extensively excised and ligated exhibited more severe lymphedema. This finding highlights a positive correlation between the extent of lymphatic damage and the severity of fluid accumulation.

Lymphatic flow was assessed using the SPY Elite^®^ Fluorescence Imaging System. In control rats, ICG and ICG-MB injections facilitated clear real-time visualization of lymphatic flow. Moreover, the nine rats in the experimental group with induced lymphedema showed a distinct interruption of lymphatic flow. Lymphatic flow appeared less distinct in the ICG-MB-injected experimental group than in the ICG-injected group at equivalent doses, likely due to reduced fluorescence intensity. However, increasing the ICG-MB injection volume restored clear visualization of lymphatic flow. These findings suggest that ICG-MB serves as a viable alternative to ICG, offering the added benefit of direct visual detectability, which could reduce reliance on specialized imaging equipment during surgical procedures.

At POD 7 and 14, the tails from 11 rats were cut 25 mm from the base. A Quantum FX micro-CT scanner was used to image the collected tail samples, and the volume of each sample was measured. Mean tail volume increased from 3992.72 ± 144.80 mm^3^ preoperatively to a maximum of 5216.71 ± 1131.88 mm^3^ at 1 week post-induction and subsequently decreased to 4614.76 ± 468.29 mm^3^ at 2 weeks. This statistically significant increase (*p* < 0.05) confirms the successful establishment of a rat tail model for lymphedema assessment.

For histopathological analysis, a tissue section was obtained 30 and 60 mm distal to the surgical site and was subjected to H&E staining. Following lymphedema induction, enhanced lymphocytic infiltration, a more intense inflammatory response, and expansion of the subcutaneous adipose layer were observed. Lymphedema led to a three- to four-fold increase in the thickness of the subcutaneous adipose layer and amplified inflammation in surrounding tissues, which may predispose to dermatitis or cellulitis under more severe conditions.

In summary, ICG, which is widely utilized for diagnosing lymphedema, emits near-infrared light beyond the visible spectrum and cannot be seen with the naked eye, necessitating the use of costly imaging equipment. In contrast, the ICG-MB reagent enables dual detection: it is fluorescent under near-infrared imaging and visually detectable due to its color. This property allows for lymphatic vessel surgery using only standard microsurgical instruments, without the need for specialized near-infrared devices. If further research supports its clinical safety and efficacy, the ICG-MB reagent could serve as a practical alternative to ICG in surgical and diagnostic settings.

However, this study has several limitations. The sample size of the experimental group was relatively small, and the observation period was limited to 2 weeks, which precludes assessment of long-term structural or functional changes. In addition, the injected volume of ICG–MB needed to be increased by approximately 1.5-fold to achieve detectability comparable to that of ICG. In the present work, our primary objective was to demonstrate the feasibility of dual-mode lymphatic visualization using ICG–MB in a rat tail lymphedema model, and therefore safety was evaluated mainly on the basis of general histologic appearance. Given the intended surgical application and the higher injected dose, future studies should incorporate comprehensive toxicity assessments, including systemic and long-term safety evaluations in larger cohorts, to support clinical translation. Furthermore, this feasibility-oriented study did not include comprehensive spectroscopic characterization of the ICG–MB conjugate, and compound analysis was restricted to stepwise yields, 1H NMR spectroscopy, optical spectra, and functional in vivo imaging performance. More extensive spectroscopic analyses would be required in dedicated chemistry-focused work to provide rigorous structural validation of the ICG–MB conjugate.

Previous experimental and clinical studies have reported that indocyanine green and methylene blue are generally well tolerated at clinically relevant doses, although dose- and context-dependent toxicity has been described in specific ocular and systemic settings. These data suggest that the individual components of ICG–MB have an acceptable safety margin in routine practice; however, the approximately 1.5-fold higher injected dose required for ICG–MB in our protocol underscores the need for future in vivo toxicity studies, including systemic and long-term evaluations, to establish a robust safety profile for this combined reagent. Integrating such dedicated toxicity assessments with the present imaging findings will be essential for supporting eventual clinical translation of ICG–MB in lymphatic surgery.

The present findings should also be interpreted in the context of anatomical differences between the rat tail model and human limbs. The rat tail is slender and relatively translucent, which facilitates visualization of superficial vessels, whereas human extremities have a much larger volume and thicker overlying soft tissues around the lymphatic channels. In this feasibility-focused study, we used the rat tail model to demonstrate that ICG–MB can provide both macroscopic color-based visualization and NIR fluorescence. Future investigations in larger animal models and in tissues that more closely resemble human limbs will be necessary to define the practical sensitivity and dosing requirements of ICG–MB under clinically relevant conditions and to better estimate its potential utility in human lymphedema surgery and related biomedical research.

A core innovation is that we focused first on modifying the fluorescent dye, rather than relying solely on developing more advanced detection hardware. We sought to improve imaging capability by altering the dye rather than optimizing instruments for ICG. We developed a synthetic dye that emits in the visible wavelength range, enabling visual observation while simultaneously allowing fluorescent staining. This is considered the most significant outcome. Fluorescent staining remains essential, as it enables preoperative mapping or staging of lymphatic vessel damage. Additionally, it supports real-time postoperative monitoring when used with noninvasive near-infrared imaging systems. Moreover, we established a rat tail model for lymphedema assessment, confirmed the potential of the new reagent ICG-MB, and finally, observed histological changes in the presence of lymphedema. Importantly, no discernible histological differences were observed between tissues from animals treated with ICG-MB and those treated with ICG alone. These results provide a foundation for future comprehensive clinical research on lymphedema and the development of treatment methods. We initially began with questions regarding the development of advanced reagents for the diagnosis and surgical treatment of lymphedema. However, since lymph nodes can also be detected using this reagent, conjugating tumor-targeting probes—currently being widely explored in cancer research—could extend their application to studies for cancer therapy. Through such integration, ICG-MB may serve as a powerful tool for tumor visualization and tracking. It may enable direct observation by the naked eye or indirect imaging via fluorescence systems.

The synthesized ICG-MB reagent enabled visual detection and near-infrared fluorescence imaging for identifying lymphedema, offering dual-mode diagnostic capability. Based on findings from our validated lymphedema animal model, we anticipate that ICG-MB may support future clinical research in lymphedema diagnosis and treatment. More broadly, this reagent may be used in the development of diagnostic and therapeutic strategies for various cancers.

## Data Availability

The original contributions presented in this study are included in the article. Further inquiries can be directed to the corresponding author.

## Abbreviations

The following abbreviations are used in this manuscript:

ICG	indocyanine green
MB	methylene blue
SD	Sprague–Dawley
IACUC	Institutional Animal Care and Use Committee
CT	Computed tomography
POD	Postoperative day
H&E	Hematoxylin and eosin
